# Measurement of China’s “External Market Provider” Role: Trade-Margin Decomposition and Gravity Determinants

**DOI:** 10.3390/e28050504

**Published:** 2026-04-30

**Authors:** Manru Zhao, Yujia Lu

**Affiliations:** School of Economics & Management, Northwest University, Chang’an District, Xi’an 710127, China; 202210014@stumail.nwu.edu.cn

**Keywords:** market provider index, Hummels-Klenow decomposition, trade-margin decomposition, destination diversification, gravity model, trade dependence

## Abstract

This paper measures China’s role as an “external market provider” by quantifying, for 168 source countries during 2001–2022, the share of each country’s exports absorbed by China and decomposing that share into extensive (product coverage), quantity, and price margins using the Hummels–Klenow framework. To characterize destination-market concentration, we construct an HHI-based network diversification indicator from export-destination shares and interpret it from a complementary information-theoretic perspective, where higher concentration corresponds to lower diversification and stronger dependence. We document the dynamics of China’s market-provision role and estimate an extended gravity-type model with country- and year-fixed effects. The results show that China’s external market-provider role expanded markedly after WTO accession, with growth driven mainly by the quantity margin and, after 2018, increasingly supported by the price margin. Economic proximity and similarity in global value-chain position are associated with stronger China-absorption shares, while greater destination concentration relative to China is associated with lower China-absorption shares. Free trade agreements are linked to stronger, more extensive, and larger margins. Robustness checks based on lagged covariates, additional controls, higher-dimensional fixed effects, Tobit estimation, and winsorization support the main findings. Overall, the paper provides a replicable framework for measuring destination-market pull and shows how China’s import-side role varies across products, regions, and development groups, while using the information-theoretic perspective as a supplementary interpretation of diversification patterns rather than as a separate empirical tool.

## 1. Introduction

In recent decades, China’s integration into the global trading system has reshaped international trade patterns, not only through its role as a major exporter but increasingly through its expanding import market. As the scale of China’s domestic economy has grown, import trade has become an important channel through which China participates in global demand allocation, absorbs foreign production, and transmits market opportunities across countries. In this context, China’s large and diversified import market has gradually evolved into a key destination for global exports, highlighting its emerging role as an “external market provider” within the international trading system. In recent years, China has become a top export destination for a growing number of economies, reflecting not only the size of its domestic market but also its increasing openness and capacity to accommodate exports from a wide range of trading partners. From an information-theoretic perspective, concentration in export dependence on a single destination market can be interpreted in terms of lower diversification and stronger destination dependence. In this study, however, this perspective is used only as a complementary interpretive lens rather than as a separate empirical measurement strategy. This conceptual linkage motivates the incorporation of entropy-linked diversification indicators in the empirical framework. Previous studies have shown that the rise in large economies can induce structural and distributional changes in global trade flows [1,2,3].

At the same time, recent developments, such as rising protectionism, trade policy uncertainty, the COVID-19 pandemic, and geopolitical conflicts, have introduced new sources of volatility into international trade [4,5,6]. Under these conditions, the stability, composition, and distributional effects of large destination markets have become increasingly important for understanding global trade resilience and adjustment. Against this backdrop, examining China’s role as an external market provider from an import-side perspective is of growing relevance. While China’s export expansion has been extensively studied, its capacity to absorb foreign exports and the structural characteristics of its import market remain comparatively underexplored. A systematic analysis of the marginal structure, dynamic evolution, and driving forces of China’s external market provision can help clarify how large destination markets shape global trade patterns and how these effects vary across countries, regions, and products. The existing literature on international trade margins and bilateral trade determinants provides a rich foundation for analyzing trade expansion mechanisms. Studies on extensive and intensive margins largely focus on exporter-side dynamics, examining how firms and countries expand along different margins in foreign markets [7,8,9,10]. Within this framework, most empirical research focuses on how exporting countries penetrate foreign markets, how firms expand along different margins, and how trade costs affect bilateral flows.

However, relatively less attention has been paid to the import-side role of destination markets as providers of external demand. Existing studies that touch upon “market provision” typically measure it using aggregate import shares or bilateral trade volumes, which obscures the underlying marginal composition and dynamic distribution of market provision. Such approaches often fail to distinguish whether a destination market’s importance arises from expanding product categories, increasing quantities, or price-related adjustments, and they rarely account for heterogeneity across regions, products, or stages of development. Moreover, much of the literature remains exporter-centric, implicitly treating destination markets as passive absorbers of exports rather than active shapers of global trade structure. From a demand-side perspective, large destination markets can influence global production patterns, trade diversification, and value chain organization [11]. Understanding how this influence operates through different trade margins is particularly important in an era characterized by heightened uncertainty and fragmented global value chains. Against this background, this paper examines China’s role as an external market provider by focusing on the marginal structure and dynamic evolution of its import market. Rather than relying on aggregate measures, we adopt a multi-dimensional approach that captures how China’s external market provision operates across product categories, quantities, and prices, and how these dimensions evolve over time and across trading partners.

Specifically, to make the analysis accessible and reusable for a broad interdisciplinary readership, we organize the empirical strategy as a transparent pipeline: (1) we construct an import-side market-share indicator that captures China’s role as an external-demand provider; (2) we decompose changes in this indicator into extensive, quantity, and price margins using a Hummels-Klenow-type framework; (3) we characterize cross-country heterogeneity and temporal evolution using distribution dynamics; and (4) we identify the correlates of China’s import-side market provision using an extended gravity specification. This end-to-end design prioritizes transparency and reproducibility, allowing the measurement framework to be readily applied to other destination markets and periods. Building on the above motivation, this study makes three specific contributions and offers an information-theoretic interpretation of HHI-based diversification measures. First, we reframe the Hummels-Klenow tripartite margin decomposition from the import (demand) side, redefining the reference set so that the extensive, quantity, and price margins capture a destination market’s absorption capacity rather than an exporter’s market penetration. Although the H-K framework itself is well established [7], its application to import-side market provision across 168 source countries over 2001–2022 is, to the best of our knowledge, new and yields insights that are not available from the conventional export-side perspective.

Second, we operationalize destination-portfolio diversification by constructing a network-distance indicator (NET) from Herfindahl-Hirschman Index (HHI) ratios and provide a complementary information-theoretic interpretation through second-order Rényi entropy. Because the standard unnormalized HHI is monotonically related to Rényi-2 entropy, the HHI-based indicators used in our gravity model can also be interpreted in terms of concentration and diversification. We do not treat entropy as a separate empirical variable; rather, it serves as a supporting conceptual perspective that helps connect the trade-economics literature on concentration indices with the broader information-theoretic discussion of diversification. Third, we document heterogeneity in the decomposed margins across continents, development groups, and BEC product categories, and identify the correlates of China’s import-side market provision using an extended gravity specification with country and year fixed effects. Unlike studies that directly introduce entropy measures as standalone empirical variables, this paper uses conventional concentration indicators as the primary analytical tools and employs the information-theoretic perspective only to support interpretation. This positioning keeps the empirical analysis centered on trade-margin decomposition and gravity determinants, while still allowing the diversification logic behind the concentration measures to be stated more clearly.

### Conceptual Framework and Testable Expectations

Our analytical logic proceeds as follows. A destination market’s “pull” on a source country’s exports can be decomposed into three channels: product coverage (whether the destination absorbs a broad or narrow set of the source’s export categories), quantity depth (how much of each product the destination absorbs relative to the world average), and price effects (whether unit values in bilateral trade differ from the world benchmark). From an information-theoretic standpoint, the degree to which a source country’s export portfolio is concentrated on a single destination (e.g., China) can be measured by the entropy of its destination-share distribution: low Shannon or Rényi entropy signals high concentration and strong dependence. We therefore expect that source countries with lower destination entropy (higher HHI) will exhibit larger MPI values, and that this relationship operates primarily through the quantity margin, because quantity deepening is the most direct manifestation of a destination’s absorptive pull. This analytical structure provides an explicit bridge between destination-market concentration in trade economics and entropy-based diversification measurement in information theory: (H1) greater economic proximity (smaller ECO distance) is associated with higher MPI, mainly through the quantity channel; (H2) greater similarity in GVC position is associated with higher MPI, because countries at similar production stages tend to have complementary intermediate-goods trade; and (H3) greater destination concentration, as captured by NET, is negatively associated with MPI.

**Figure 1:**
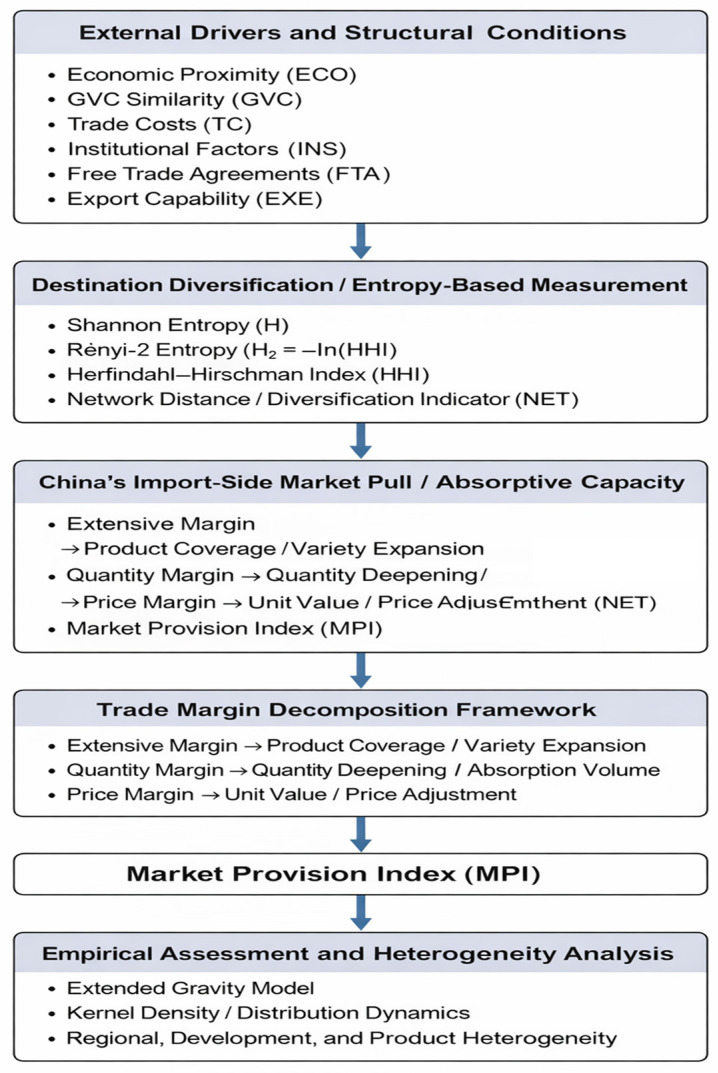
Conceptual framework of China’s imports.

The gravity model in Section 3.4 operationalizes these expectations. As shown in Figure 1, the framework illustrates how structural trade factors and entropy-based destination diversification jointly influence China’s absorptive market pull, which is decomposed into extensive, quantity, and price margins and empirically assessed through the Market Provision Index (MPI) and extended gravity model. The remainder of this paper is organized as follows. Section 2 reviews the relevant literature on trade margins, market provision, and bilateral trade determinants. Section 3 presents the research design, including the tripartite marginal decomposition framework, the entropy-based interpretation of destination diversification, kernel density estimation [12], the gravity model specification, and data sources. Section 4 reports the measurement results of China’s external market provision and its ternary margins. Section 5 analyzes the correlates and heterogeneity across regions, countries, and products. Section 6 concludes with a discussion of the main findings and their implications. We first measure China’s import-side market share and decompose its changes into extensive, quantity, and price margins. We then examine cross-country heterogeneity via distribution dynamics, and finally estimate an extended gravity model to assess the drivers of China’s external market-provider status.

## 2. Literature Review

The academic literature on international trade margins, market provision, and the determinants of bilateral trade flows provides the theoretical foundation for this study. We organize the literature review into four main streams: (1) trade margin decomposition methods, (2) gravity model applications, (3) China’s trade patterns and market role, and (4) correlates of bilateral trade.

### 2.1. Trade Margin Decomposition Methods

The decomposition of trade flows into extensive and intensive margins has become a standard approach in international trade research [13]. The seminal work by Hummels and Klenow [7] introduced the tripartite decomposition framework that separates a country’s exports into variety (extensive margin), quantity, and price components. This methodology has been widely adopted and extended in subsequent studies. Chaney [8] developed a theoretical model showing how trade costs affect the extensive and intensive margins differently, with the extensive margin being more sensitive to variable trade costs. Helpman et al. [9] further refined the estimation of trade flows by explicitly modeling the selection of trading partners and the volume of trade. The application of margin decomposition to understand trade dynamics has yielded important insights. Bernard et al. [14] extended the framework to multiproduct firms, showing how trade liberalization affects product scope decisions. Feenstra and Romalis [15] demonstrated that international prices are heavily influenced by quality, developing an extended monopolistic competition framework where firms simultaneously choose price and quality. Amiti and Freund [16] applied these methods to analyze China’s export growth, finding that despite dramatic structural changes, the intensive margin (existing products to existing markets) accounted for the bulk of export growth [17]. Badinger and Türkcan [18] assessed the trade effects of currency unions through margin decomposition, relating disaggregated estimates to the elasticity of substitution.

### 2.2. Gravity Model Applications

The gravity model has been extensively used in international trade research for its considerable empirical robustness and explanatory power [13,19]. Anderson and van Wincoop [20,21] provided theoretical foundations by deriving the gravity equation from a general equilibrium trade model, emphasizing the importance of multilateral resistance terms. Deardorff [22] demonstrated that the gravity equation can be derived from both Heckscher-Ohlin and other trade models, establishing its theoretical versatility. Recent advances have refined gravity model applications. Baier and Bergstrand [23] addressed endogeneity concerns in estimating FTA effects, finding that properly specified gravity models show FTAs approximately double bilateral trade after 10 years. Baldwin and Taglioni [24] identified common pitfalls in gravity equation estimation, including the “gold medal mistake” of omitting multilateral resistance terms.

### 2.3. China’s Trade Patterns and Market Role

China’s integration into the global economy has attracted substantial research attention. Du and Lu [25] examined China’s international trade development, noting that the astonishing surge in trade resulted from comprehensive economic reforms and was further bolstered by WTO accession in 2001. Manova and Yu [26] investigated how Chinese firms export, distinguishing between processing and ordinary trade with financial frictions. The role of imports in China’s economic development has also been examined. Goldberg et al. [27] used detailed trade and firm-level data from India to show substantial gains from trade through access to new imported inputs, with implications for China’s import-driven productivity growth. Mo et al. [28] provided direct evidence from Chinese manufacturing firms, demonstrating that what firms import matters significantly for productivity growth. Bastos [29] analyzed the exposure of Belt and Road economies to China trade shocks, highlighting the growing importance of China as an export destination for developing countries [30].

### 2.4. Correlates of Bilateral Trade

The literature has identified numerous factors affecting bilateral trade flows. First, regarding economic similarity and demand patterns, Linder [31] proposed the similar demand hypothesis, suggesting that countries with similar per capita incomes trade more intensively. This theory provides a framework for understanding why economic distance affects trade patterns. Second, global value chain (GVC) participation has emerged as a crucial determinant of trade. Koopman et al. [32] developed methods for tracing value-added and identifying double counting in gross exports, enabling more accurate measurement of GVC positions. Johnson and Noguera [33] proposed accounting frameworks for production sharing and trade in value added. Antràs and Chor [34] provided a comprehensive review of GVC research, highlighting how a country’s position in value chains affects its trade patterns. Meng et al. [35] specifically examined China’s trade in value added using both national accounting and production approaches. Third, institutional factors significantly influence trade flows. François and Manchin [36] examined how infrastructure and institutional quality affect bilateral trade, emphasizing threshold effects. De Groot et al. [37] analyzed the institutional determinants of bilateral trade patterns using gravity models.

Similarly, Liu et al. [38] provided evidence on how cultural and institutional distance affect China’s trade with Belt and Road countries. Álvarez et al. [39] examined whether institutional quality matters for trade across different sectors. Fourth, exchange rate dynamics affect trade volumes. The literature documents significant effects of exchange rate volatility on bilateral trade flows [40,41,42], with the direction and magnitude depending on the nature of volatility and invoicing practices [43]. Fifth, regional trade agreements and integration have received considerable attention. Studies have documented trade creation and diversion effects of the ASEAN–China Free Trade Area [44] and assessed the potential of the Regional Comprehensive Economic Partnership (RCEP) [45] for promoting regional integration [46,47,48]. Sixth, trade network effects have been studied using network analysis methods. Fagiolo et al. [49] analyzed the evolution of the world trade web using weighted-network analysis. Deguchi et al. [50] identified hubs and authorities in the world trade network, providing insights into the structure of global trade relationships.

### 2.5. Research Gaps

Despite the rich literature on trade margins, gravity models, and China’s trade patterns, two gaps remain. First, the H–K margin decomposition has been applied almost exclusively from the export side, leaving the import-side (demand-side) decomposition largely unexplored. Second, while concentration and diversification indices such as HHI are routinely used in trade-network studies, their connection to information-theoretic entropy measures is seldom made explicit, limiting cross-disciplinary comparability. This paper addresses both gaps by applying the H-K decomposition from the import side and by embedding an HHI-based diversification indicator—interpreted through the lens of Renyi entropy—in the gravity framework.

## 3. Research Design

### 3.1. Tripartite Marginal Decomposition Framework

Previous studies have defined the proportion of Japan’s imports from China to China’s total exports to the world as Japan’s “Market Provider Index” (MPI) for China, to measure Japan’s role as an “external market provider” to China [51], as shown in Equation (1):

(1) $${MPI}_{ij}=\frac{\sum_{k\in U_{ij}}p_{ijk}q_{ijk}}{\sum_{k\in U_{iw}}p_{iwk}q_{iwk}}$$

In the equation, i, j, and w refer to the exporting country (i.e., China’s import source country), the importing country (specifically China), and the world, respectively. k is the six-digit product code of the HS96 version. $K_{\mathrm{ij}}$ and $K_{\mathrm{iw}}$ represent the export product sets of country i to country j and to the world, respectively. $p_{\mathrm{ij},k}$ and $p_{\mathrm{iw},k}$ denote the corresponding unit export prices. $q_{\mathrm{ij},k}$ and $q_{\mathrm{iw},k}$ denote the export quantity of product k from country i to country j and from country i to the world, respectively. The export value of product k from country i to country j is therefore $x_{\mathrm{ij},k}\;=\;p_{\mathrm{ij},k}\;\times {\;q}_{\mathrm{ij},k}$, and similarly $x_{\mathrm{iw},k}\;={\;p}_{\mathrm{iw},k}\;\times \;q_{\mathrm{iw},k}$ for exports to the world. Equation (1) can thus be read as the ratio of country i’s total export value to China (j) over its total export value to the world (w), both computed as the sum of price times quantity across all products. As shown in Equations (2) and (3): Using the H-K tripartite marginal decomposition method [7], which has been widely applied in international trade research [8,9], MPI is decomposed into the product category marginal (EM), quantity marginal (QM), and price marginal (PM) products, representing the breadth of category overlap, the depth of quantity coverage, and the height of price reach of country j’s exports to country i relative to the global average:

(2) $${\mathrm{MPI}}_{\mathrm{ij}}\;=\;EM_{\mathrm{ij}}\;\times \;QM_{\mathrm{ij}\;}\times \;PM_{\mathrm{ij}}$$

(3) $$EM_{\mathrm{ij}}=\frac{\sum_{k\in U_{\mathrm{ij}}}p_{\mathrm{iwk}}q_{\mathrm{iwk}}}{\sum_{k\in U_{\mathrm{iw}}}p_{\mathrm{iwk}}q_{\mathrm{iwk}}},\;QM_{\mathrm{ij}}={\prod_{k\in U_{\mathrm{ij}}}\left(\frac{q_{\mathrm{ijk}}}{q_{\mathrm{iwk}}}\right)}^{\omega_{\mathrm{ijk}}},\;PM_{\mathrm{ij}}={\prod_{k\in U_{\mathrm{ij}}}\left(\frac{p_{\mathrm{ijk}}}{p_{\mathrm{iwk}}}\right)}^{\omega_{\mathrm{ijk}}}$$

As shown in Equation (4), Here, the weight $\omega_{ijk}$ is the logarithmic average of the export share of product k from country i to country j ($S_{ijk}$) and the export share of product k from country i to the world ($S_{iwk}$):

(4) $$\omega_{\mathrm{ijk}}\;=\;\frac{\left(S_{\mathrm{ijk}}-S_{\mathrm{iwk}})/(\ln S_{\mathrm{ijk}}-\ln S_{\mathrm{iwk}}\right)}{\sum_{k\in U_{\mathrm{ij}}}[(S_{\mathrm{ijk}}-S_{\mathrm{iwk}})/(\ln S_{\mathrm{ijk}}-\ln S_{\mathrm{iwk}})]},\;S_{\mathrm{ijk}}\;=\;\frac{p_{\mathrm{ijk}}q_{\mathrm{ijk}}}{\sum_{k\in U_{i}}T_{\mathrm{ik}}\;q_{\mathrm{ijk}}},{\;S}_{iwk}\;=\;\frac{p_{\mathrm{iwk}}q_{\mathrm{iwk}}}{\sum_{k\in U_{\mathrm{ij}}}p_{\mathrm{iwk}}q_{\mathrm{iwk}}}$$

As shown in Equations (5)–(8), Drawing on Shi [52] methodology of aggregating three-way margins across countries for inter temporal comparisons, $M_{mj}$ denotes the set of import source countries for country j, while the weight aij represents the market share of country i in j’s total imports. The superscripts 0 and t denote the base period and current period, respectively. By taking natural logarithms and dividing by the interval years, intertemporal comparisons can be conducted:

(5) $$EM_{j}\;=\;\prod_{i\in M_{\mathrm{mj}}}(EM_{\mathrm{ij}}{)}^{a_{\mathrm{ij}}},\;QM_{j}\;=\;\prod_{i\in M_{\mathrm{mj}}}(QM_{\mathrm{ij}}{)}^{a_{\mathrm{ij}}},\;PM_{j}\;=\;\prod_{i\in M_{\mathrm{mj}}}(PM_{\mathrm{ij}}{)}^{a_{\mathrm{ij}}}$$

(6) $$\frac{{\mathrm{MPI}}_{j}^{t}}{{\mathrm{MPI}}_{j}^{0}}=\frac{EM_{j}^{t}}{EM_{j}^{0}}\;\times \;\frac{QM_{j}^{t}}{QM_{j}^{0}}\;\times \;\frac{PM_{j}^{t}}{PM_{j}^{0}}$$

(7) $$G_{\mathrm{MPI}}=g_{\mathrm{EM}}+g_{\mathrm{QM}}+g_{\mathrm{PM}\;}$$

(8) $$C_{\mathrm{EM}}=\frac{g_{\mathrm{EM}}}{g_{\mathrm{MPI}}}\;\times \;100,\;C_{\mathrm{QM}}=\frac{g_{\mathrm{QM}}}{g_{\mathrm{MPI}}}\;\times \;100,\;C_{\mathrm{PM}}=\frac{g_{\mathrm{PM}}}{g_{\mathrm{MPI}}}\;\times \;100\;$$

### 3.2. Entropy-Based Interpretation of Destination Diversification

To connect the trade-margin decomposition framework with information-theoretic ideas of diversification, we provide a complementary interpretation of destination concentration using entropy-related concepts [53]. Let $s_{\mathrm{jd}}$ denote the share of country j’s total exports directed to destination d, with the shares summing to one over all destinations $d\;\in \;D_{j}$. The Shannon entropy of this destination distribution is:

(9) $$H_{1}(j)=-\sum_{d\in D_{j}}s_{jd}\ln s_{\mathrm{jd}}$$

Shannon entropy reaches its maximum ($\ln|D_{j}|$) when exports are evenly distributed across destinations and falls as exports become concentrated on fewer markets. Accordingly, higher entropy indicates greater diversification, whereas lower entropy indicates stronger destination dependence. A related measure is the second-order Rényi entropy. For α=2, the second-order Rényi entropy is:

(10) $$H_{2}(j)=-\ln\left(\sum_{d\in D_{j}}s_{jd}^{2}\right)=-\ln({\mathrm{HHI}}_{j})$$

where ${\mathrm{HHI}}_{j}\;=\sum_{d\in D_{j}}s_{\mathrm{jd}}^{2}$ is the Herfindahl–Hirschman Index of country j’s destination concentration. Because −ln(⋅) is a strictly decreasing function, higher HHI (greater concentration) maps one-to-one to lower Rényi-2 entropy (less diversification). The ordering property $H_{1}\geq H_{2}$ (with equality only when all shares are equal) implies that Rényi-2 provides a more conservative, concentration-sensitive measure of diversification than Shannon entropy. We emphasize that the contribution of the entropy framework in this paper is interpretive rather than introducing a new empirical variable. We emphasize that the empirical analysis uses the standard unnormalized Herfindahl-Hirschman Index, defined as ${HHI}_{i}=\sum_{d\in D_{j}}s_{\mathrm{jd}}^{2}$. Accordingly, the identity $H_{2}(i)=-\ln(HHI_{i})$ applies directly to the NET indicator used in the regression analysis. Entropy is therefore used as an information-theoretic interpretation of the HHI-based concentration measure, rather than as a separate empirical variable. This entropy lens provides a single, interpretable scale on which concentration, diversification, and dependence can be compared across countries and over time. In our empirical design, we exploit this monotonic relationship in two ways. First, the network-distance indicator ${\mathrm{NET}}_{\mathrm{ijt}}$ used in the gravity model (Section 3.4) is constructed from HHI ratios, so it can be directly interpreted as measuring the relative Rényi-2 entropy gap between country i’s destination portfolio and China’s import-source portfolio.

A higher NET indicates greater destination concentration of country i relative to China, i.e., lower destination diversification and lower Rényi-2 entropy. We therefore expect NET to be negatively associated with MPI if countries with more concentrated export portfolios are less dependent on China than those with broader destination diversification. Second, we use the entropy framework to interpret the distribution dynamics of MPI (Section 3.3): rightward shifts and widening spreads in the MPI distribution correspond to a decrease in the average destination entropy across source countries, signaling growing concentration on China as a destination market. This formalization ensures that the entropy concepts are not merely terminological but are embedded in both the measurement (via HHI-to-entropy mapping) and the interpretation (via diversification-dependence logic) of the empirical results. We do not claim that entropy introduces a new empirical variable beyond HHI; rather, the entropy lens provides a complementary interpretive perspective in which concentration, diversification, and dependence can be compared on a well-defined information-theoretic scale, connecting our trade-specific indicators to the broader literature on entropy in complex systems. The entropy-based lens is particularly useful because it provides a single, interpretable scale on which concentration, diversification, and dependence can be compared across countries and over time, and it connects our trade-specific indicators to a broader literature on entropy in complex systems [49,50].

### 3.3. Kernel Density Estimation

As shown in Equation (11), using the kernel density estimation method to present China’s global position as an “external market provider” and the dynamic evolution of its ternary marginal distribution [51], the specific formula is as follows:

(11) $$K(x)\;=\;\frac{1}{\sqrt{2\pi }}\exp\left(-\frac{x^{2}}{2}\right),\;F(x)\;=\;\frac{1}{\mathrm{nh}}\sum_{i=1}^{n}K\left(\frac{X_{i}-x}{h}\right)$$

Here, n denotes the number of observations for each variable, Xi represents independent and identically distributed trivariate marginal observations, x denotes the mean of the observations, h is the optimal window width determined by Silverman [13], and K (·) denotes the Gaussian kernel density function.

### 3.4. Construction of the Gravity Model

The gravity model has been extensively used in international trade research due to its considerable empirical robustness and explanatory power [13,21]. Following the extended gravity model framework widely adopted in the literature [20,23,24], we construct the following specification for China’s external market provision and its three-way marginal correlates. Our baseline specification is a log-linear OLS model with country and year fixed effects, where the dependent variable is $\ln({\mathrm{MPI}}_{\mathrm{ijt}})$ (or ln(EM), ln(QM), ln(PM) for the margin-specific regressions). We note that MPI is bounded between 0 and 1, so its logarithm takes negative values; this is algebraically valid and does not affect estimation consistency under standard regularity conditions. As robustness checks, we employ five alternative specifications to assess the sensitivity of the baseline results: (1) lagged covariates to address potential simultaneity, (2) additional controls for resource endowments, (3) higher-dimensional fixed effects, (4) Tobit estimation to account for the bounded nature of MPI, and (5) winsorization to mitigate the influence of outliers. The Tobit specification is particularly relevant because MPI is bounded between 0 and 1; Tobit explicitly models this censoring and provides consistent estimates under the assumption of a latent normally distributed variable.

Following the extended gravity framework, we estimate the determinants of China’s external market provision using a common specification. Importantly, China’s market provision index (MPI) is constructed from the tripartite decomposition into the category margin (EM), quantity margin (QM), and price margin (PM). Therefore, rather than mechanically regressing MPI on its own components, we estimate Equation (12) separately for four dependent variables ${\;Y}_{\mathrm{ijt}\;}\in \;\{{\mathrm{MPI}}_{\mathrm{ijt}},{\;\mathrm{EM}}_{\mathrm{ijt}},\;QM_{\mathrm{ijt}},\;PM_{\mathrm{ijt}}\}$. This design allows us to identify whether each gravity factor operates primarily through product-category coverage (EM), quantity deepening (QM), or unit-value/price (PM), while controlling for country fixed effects and year fixed effects to account for multilateral resistance.

(12) $${\mathrm{ECO}}_{\mathrm{ijt}}=\frac{{\left(Y_{i}-Y_{j}\right)}^{2}}{Y_{i}Y_{j}}$$

${\mathrm{ECO}}_{\mathrm{ijt}}$ represents the international division of labor status distance. Global value chain participation has become a crucial determinant of trade patterns [32,33,34]. We measure the ECO position distance as the absolute value of the difference in China’s and source countries’ value chain position indices ($ECO_{Posiit}$) [35]:

(13) $${\mathrm{GVC}}_{\mathrm{Posiit}}\;=\;\ln\left(1+\frac{{\mathrm{IVA}}_{\mathrm{it}}}{{\mathrm{DVA}}_{\mathrm{it}}+{\mathrm{FVA}}_{\mathrm{it}}}\right)-\ln\left(1+\frac{{\mathrm{FVA}}_{\mathrm{it}}}{{\mathrm{DVA}}_{\mathrm{it}}+{\mathrm{FVA}}_{\mathrm{it}}}\right)$$

As shown in Equation (13), where DVA, FVA, and IVA represent domestic value added, foreign value added, and indirect value added respectively.

(14) $$TC_{\mathrm{ijt}}=\frac{EX_{\mathrm{ijt}}-IM_{\mathrm{ijt}}}{EX_{\mathrm{ijt}}+IM_{\mathrm{ijt}}}$$

As shown in Equation (14), $TC_{\mathrm{ijt}}$ represents the comparative advantage to China measured by the trade competitiveness index, following the revealed comparative advantage literature [54,55]:

(15) $$N{ET}_{ijt}=HHI_{i}/HHI_{j},\;HHI_{i}=\sum_{d\in D_{i}}{s_{id}}^{2}$$

As shown in Equation (15), ${\mathrm{NET}}_{\mathrm{ijt}}$ represents the third-market (network) diversification distance, reflecting the “third-party effect”. Trade network effects have been shown to influence bilateral trade patterns [49,50]. We measure diversification using the Herfindahl-Hirschman Index (HHI) computed over each country’s export-destination shares, where $D_{i}$ is the set of all export destinations of country i and $s_{\mathrm{id}}$ is the share of country i’s total exports going to destination d. As shown in Section 3.2, ${\mathrm{HHI}}_{i}$ is monotonically linked to second-order Rényi entropy via $H_{2}\left(i\right)=-\ln({\mathrm{HHI}}_{i})$. We define ${\mathrm{NET}}_{\mathrm{ijt}}$ as the ratio of destination-market dispersion between country i and China (country j): A higher NET indicates greater destination concentration of country i relative to China, i.e., lower diversification and lower entropy. Accordingly, we expect NET to be negatively associated with MPI if exporters with more concentrated destination portfolios are less dependent on China than exporters with broader diversification.

(16) $${\mathrm{EXE}}_{\mathrm{ijt}}=\left(\frac{{\mathrm{nom}}_{\mathrm{it}}}{{\mathrm{def}}_{\mathrm{it}}}\right)\left(\frac{{\mathrm{nom}}_{\mathrm{jt}}}{{\mathrm{def}}_{\mathrm{jt}}}\right)$$

As shown in Equation (16), ${\mathrm{EXE}}_{\mathrm{ijt}}$ represents the relative value of the RMB to measure purchasing power. Exchange rate dynamics have been shown to affect trade volumes, with mixed effects depending on the nature of volatility [40,41,42]:

(17) $${\mathrm{INS}}_{\mathrm{ijt}}\;=\;\sum_{k=1}^{n}\left[{\left(I_{ik}-I_{jk}\right)}^{2}/v_{k}\right]/n$$

As shown in Equation (17), ${\mathrm{INS}}_{\mathrm{ijt}}$ represents the institutional distance between China and source countries. Institutional factors significantly influence trade flows [36,37,38,39]:

Additionally, the model sets a dummy variable (${\mathrm{FTA}}_{\mathrm{ijt}}$) based on the effective year to indicate whether a free trade agreement has been signed with China. The effects of FTAs on bilateral trade have been extensively studied [23,44,56]. We also include controls for country and time fixed effects to address multilateral resistance [21,24].

Multicollinearity diagnostics. Before estimation, we examined pairwise correlations among the key regressors (ECO, GVC, TC, NET, INS, EXE, FTA). The highest pairwise correlation is between ECO (economic proximity) and TC (trade competitiveness), at approximately 0.45, which is below conventional concern thresholds. To assess potential multicollinearity among explanatory variables, Variance Inflation Factors (VIFs) were computed for all regressors in the baseline model. As reported in Table 1, all VIF values remain below the threshold value of 5, with a mean VIF of 2.20, suggesting that multicollinearity does not pose a serious concern for estimation reliability. In addition, the inclusion of country fixed effects absorbs time-invariant bilateral characteristics, further mitigating collinearity arising from omitted structural variables.

Endogeneity considerations. We acknowledge potential endogeneity in several regressors. For instance, countries that already trade heavily with China may be more likely to sign FTAs (reverse causality in ${\mathrm{FTA}}_{\mathrm{ijt}}$), and unobserved bilateral political relationships could drive both trade concentration and some of our regressors (omitted variable bias). Our panel design with country and year fixed effects partially addresses these concerns by controlling for time-invariant confounders and common global shocks. However, we do not claim strict causal identification; the gravity estimates should be interpreted as conditional associations. As an additional robustness check, we lag the key time-varying regressors (ECO, GVC, TC, NET, EXE, INS) by one period to partially address simultaneity concerns. The results, reported in the robustness section, are qualitatively similar to the baseline estimates.

### 3.5. Data Sources and Explanations

The data in this paper are sourced from the China Free Trade Zone Service Network. The research period spans from 2001 to 2022, covering 168 source countries (regions) that China imported from continuously during this period. Additional supporting data, such as macroeconomic indicators, regional statistics, and control variables, were obtained from relevant official statistical yearbooks and government databases. The study is divided into three phases based on key milestones: China’s WTO accession in 2001, the 18th National Congress of the Communist Party of China in 2012, and the Boao Forum in 2018. The first phase (2001–2012) is the “import service export” phase; the second phase (2013–2018) is the “import-export parity” phase; and the third phase (2019–2022) is the “proactive import expansion” phase. In accordance with the WITS database’s trade classification conversion table, the six-digit HS codes in the CEPII-BACI database were consolidated into primary goods, intermediate goods, capital goods, and consumer goods under the BEC Rev.4 standard (Table 2).

## 4. Results of China’s External Market Share

### 4.1. Overall Trends in China’s External Market Provision and Three-Way Margins

**Figure 2:**
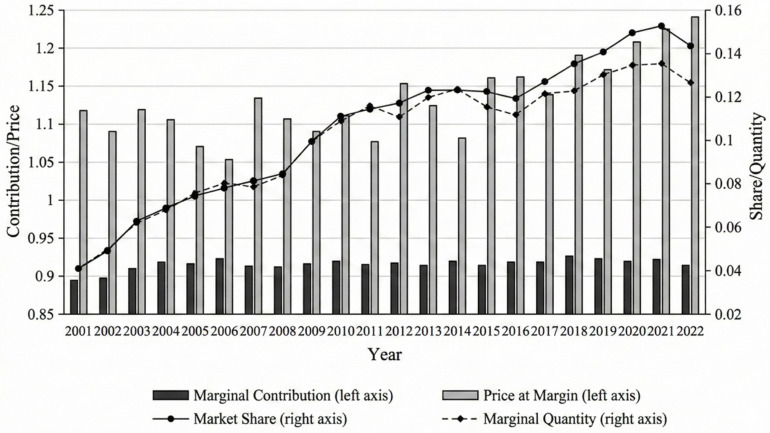
China’s share and three-way marginal fluctuation trends in the global market.

Figure 2 reports China’s import-side market provision index (MPI) and its Hummels-Klenow-type tripartite margins from 2001 to 2022. Over the sample period, China’s MPI rises markedly, indicating a sustained expansion of China’s role as a destination market that absorbs foreign exports. The series exhibits only short episodes of contraction, suggesting that the long-run trend is dominated by an expansion in China’s import-side market share rather than by persistent reversals. The tripartite decomposition highlights three structural features. First, the category (extensive) margin (EM) remains persistently high, implying that China’s import demand overlaps with a broad set of world product categories for most source countries. Second, the quantity margin (QM) increases steadily and co-moves closely with MPI, indicating that quantitative deepening is the primary contributor to China’s rising import-side market provision. Third, the price (unit-value) margin (PM) stays above one in all years and shows pronounced fluctuations, with an upward tendency in the later period. Because unit values may reflect quality composition, market power, and/or short-run price shocks [57], we interpret PM as a comprehensive price-related margin rather than a pure measure of quality. Taken together, these patterns suggest that China’s external market provision is characterized by (1) a high and stable breadth of product coverage, (2) sustained deepening of quantities as the dominant expansion mode, and (3) increasingly important price-related dynamics in the more recent years.

To contextualize China’s trajectory, we briefly compare its MPI dynamics with those of the United States and the European Union as alternative large destination markets. Over the same 2001–2022 period, the US import-side MPI declined slightly from approximately 15% to 12%, reflecting a gradual loss in the US’s relative absorptive pull as emerging markets diversified their export destinations. The EU’s aggregate MPI remained relatively stable at around 18–20%, consistent with its role as a mature, broad-based import market. By contrast, China’s MPI rose from approximately 5% to over 14%, making it the fastest-growing large destination market in relative terms. This divergence underscores that China’s rising role as an external market provider is not simply a reflection of global trade growth but represents a structural reallocation of destination-market share, primarily at the expense of developed-economy importers. A full comparative analysis using the tripartite decomposition for the US and EU is beyond the scope of this paper but represents a natural extension of the framework presented here.

### 4.2. Distribution Dynamics

**Figure 3:**
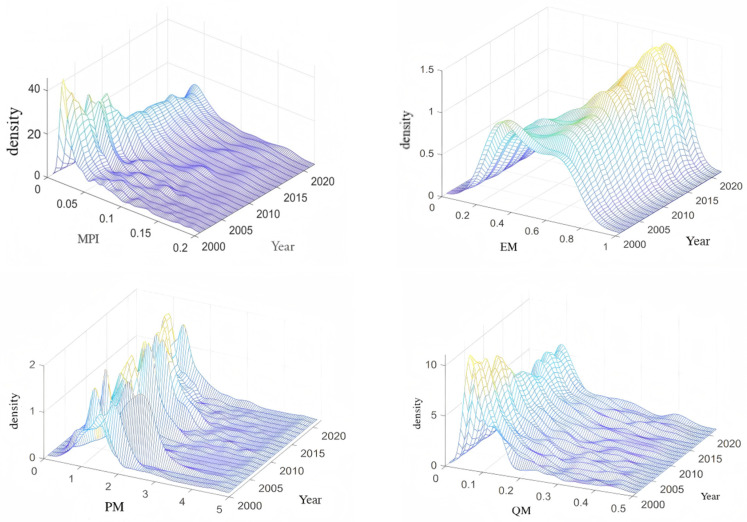
3D kernel density map of China’s share and marginal characteristics in the external market.

Figure 3 presents kernel-density evidence on the cross-country distribution of MPI and each margin. The distribution of MPI displays a rightward shift over time together with a widening spread and an increasingly visible right tail. In economic terms, the rightward shift indicates that China’s import-side market provision has strengthened on average: a growing number of source countries direct a larger share of their exports to China. The widening spread and the emerging right tail signal increasing polarization in partner-country dependence, with a subset of economies (predominantly resource-rich developing countries in Africa, South America, and Oceania) becoming substantially more reliant on China as a destination market, while others maintain more diversified export portfolios. From an entropy perspective, this distributional evolution corresponds to a decline in the average destination entropy across source countries: more countries are shifting probability mass toward a single destination (China), which lowers their Shannon and Rényi-2 entropy. We note that kernel density analysis is descriptive and does not establish causal relationships; it serves to characterize the cross-country heterogeneity that the subsequent gravity analysis (Section 5) aims to explain. The EM distribution evolves from a more dispersed (and occasionally multi-peaked) pattern toward a more concentrated mass at high values, consistent with convergence in category coverage as China’s import demand becomes broad-based across partners.

By contrast, the QM distribution resembles MPI: it shifts rightward and becomes more dispersed, reinforcing the view that quantitative deepening is the key driver of both average expansion and cross-country differentiation. Finally, the PM distribution exhibits recurrent single- and double-peak patterns and shows greater dispersion and a more pronounced right tail in the post-2018 period, suggesting increased polarization in price-related margins across source countries. Overall, the distribution dynamics indicate that China’s rising role as an external market provider is accompanied by widening asymmetries in partner-country dependence, primarily through quantity deepening and, increasingly, through price-related dynamics. The polarization observed in the PM distribution after 2018, in particular, may reflect divergent pricing strategies, quality-composition shifts, or terms-of-trade effects linked to the US–China trade frictions and pandemic-related supply disruptions. These distributional patterns motivate the gravity analysis in Section 5, where we examine which country-level and bilateral factors are associated with higher or lower China-absorption shares across the three margins. From a distributional perspective, the right-skewed density curves indicate that a relatively small number of countries exhibit strong dependence on China’s import market, whereas the majority maintain moderate exposure. Over time, shifts in the density peak and changes in dispersion suggest evolving convergence and divergence patterns in destination dependence.

Interpreted through the entropy framework developed in Section 3.2, a narrowing distribution implies greater similarity in destination portfolio diversification across countries, while widening tails indicate increasing heterogeneity in destination concentration.

### 4.3. Marginal Contribution Rate

Table 3 quantifies how each margin contributes to the growth of MPI. Over the full sample, MPI grows at an average annual rate of 5.98%, and the quantity margin accounts for the majority of this expansion (89.93%), while the price margin contributes a smaller but non-negligible share (8.37%). The category margin contributes little on average (1.70%), consistent with EM being already high and relatively stable. The phase decomposition further reveals a structural transition. While China’s MPI level continues to rise across phases, the growth rate declines, indicating maturation in China’s role as a destination market. Importantly, the contribution of PM rises sharply in the most recent phase (2019–2022), while the contribution of QM declines, implying a shift from a predominantly “quantity-driven” expansion toward a pattern in which quantity and price-related dynamics jointly shape market provision.

Product-level heterogeneity (Table 4) shows that China’s market provision is stronger for primary goods and intermediate goods than for consumer goods. The quantity margin dominates expansion across product groups, but PM plays a more prominent role for intermediate and consumer goods, consistent with stronger unit-value dynamics in these categories. At the same time, the comparatively modest MPI for consumer goods indicates that China’s import-side market provision is less pronounced in consumption-oriented products than in production-related inputs.

Continental heterogeneity (Table 5) demonstrates that China’s external market provision differs substantially across regions. China’s average MPI is higher and grows faster for Oceania, Africa, and South America than for Europe and North America. The margin decomposition suggests that these regional patterns are largely driven by stronger quantity deepening in regions where exporters may rely more heavily on China’s demand, whereas PM tends to be higher for developed regions, consistent with higher unit values in imports sourced from Europe and North America.

Table 6 compares developed and developing source countries. China’s MPI is higher and grows faster for developing countries, with expansion primarily driven by the quantity margin. For developed countries, PM contributes relatively more to MPI growth, indicating that price-related dynamics matter more in China’s imports from advanced economies.

### 4.4. Correlation Analysis

#### 4.4.1. Benchmark Regression

This section examines the determinants of China’s external market provision and its three margins using the gravity specification in Equation (12). Because MPI is constructed from the tripartite decomposition into EM, QM, and PM, regressing MPI on these three components would be tautological. We therefore estimate the same set of gravity covariates separately for four dependent variables: MPI, EM, QM, and PM. Table 7 reports the benchmark results with country fixed effects, year fixed effects, and clustered robust standard errors. Comparing coefficients across columns allows us to identify whether each gravity factor mainly operates through product-category coverage (EM), quantity deepening (QM), or price/unit-value adjustment (PM). The benchmark estimates show that gravity-type frictions are systematically associated with China’s external market provision, which is broadly consistent with the literature on GVC participation and bilateral trade patterns [32,34]. The impact of network distance on China’s import share is negative. Given that higher NET denotes greater destination concentration rather than greater diversification, this result suggests that exporters with more concentrated destination portfolios tend to exhibit lower China-absorption shares. The institutional distance and bilateral exchange rates were not significant, which aligns with the mixed findings in the literature on exchange rate effects [40].

The implementation of FTA significantly promoted China’s status as an external market provider, consistent with the trade creation effects documented in Baier and Bergstrand [23] and Yang and Martínez-Zarzoso [44]. Economic distance ${ECO}_{ijt}$ is associated with a lower ${MPI}_{ijt}$ (coefficient: −0.017, *p* < 0.10), and the margin regressions indicate that this effect is mainly transmitted through the QM (quantity) channel, where the coefficient (−0.022) is statistically significant at the 1% level, while the EM and PM coefficients are not significant. This is consistent with hypothesis H1 and the theory of similar demand [31]: countries that are economically closer to China absorb larger quantities of Chinese imports per product category. Similarly, a larger GVC-position distance ${GVC}_{ijt}$ is associated with a decline in ${\mathrm{MPI}}_{ijt}$ (coefficient: −0.085, *p* < 0.05), with the dominant contribution coming from the QM (quantity) margin (coefficient: −0.268, *p* < 0.01). This supports hypothesis H2: countries at similar stages of production to China engage in more intensive intermediate-goods trade, deepening quantity-based absorption. Comparative advantage ${\mathrm{TC}}_{ijt}$ is positively related to overall provision and is most strongly reflected in the PM (price) margin (coefficient: 0.187, *p* < 0.01), suggesting that countries with stronger revealed comparative advantage relative to China tend to export at higher unit values, consistent with quality differentiation and specialization in higher-value product segments.

Network distance ${\mathrm{NET}}_{ijt}$ primarily operates through the EM (extensive) margin (coefficient: −0.094, *p* < 0.01). Because higher NET denotes greater destination concentration rather than greater diversification, the negative coefficient implies that exporters with more concentrated destination portfolios tend to trade fewer product categories with China and exhibit lower China-absorption shares. This result is consistent with the interpretation of NET as an entropy-linked measure of destination concentration relative to China. Finally, ${\mathrm{FTA}}_{ijt}$ is associated with higher ${\mathrm{MPI}}_{ijt}$ (coefficient: 0.027, *p* < 0.01), with the largest significant coefficient observed in the QM (quantity) regression (coefficient: 0.024, *p* < 0.05), implying that trade agreements primarily expand China’s market provision through deeper quantity absorption of existing and new products.

#### 4.4.2. Robustness Checks

Table 8 provides a battery of robustness exercises designed to assess the sensitivity of the baseline results from multiple angles. Model 1 uses lagged covariates to address potential simultaneity between the regressors and MPI. Model 2 adds controls for resource endowments to account for omitted commodity-specific drivers. Model 3 introduces higher-dimensional fixed effects to absorb additional unobserved heterogeneity. Model 4 employs Tobit estimation, which explicitly accounts for the bounded nature of MPI (constrained between 0 and 1) and provides a natural alternative to fractional response models for censored outcomes. Model 5 applies winsorization to mitigate the potential influence of extreme outliers. Across all five specifications, the key signs and the main inferences remain stable: economic and value-chain proximity tend to strengthen China’s market provision, comparative advantage increases China’s MPI, network distance reduces reliance on China’s market, and FTAs are generally associated with higher China-oriented market shares. These results support the robustness of the benchmark findings to alternative specifications and data treatments.

### 4.5. Heterogeneity Analysis

#### 4.5.1. Country-Specific Heterogeneity

As shown in Table 9, the effects of various factors on MPI are more significant in developing countries than in developed countries. Economic distance and value chain status gap have significantly negative impacts on China’s market share in developing countries but are not significant in developed countries. The comparative advantage with China is significantly positive only in developing countries. Network distance has a significantly negative impact on China’s MPI from developing countries. The positive effect of FTA entry on China’s MPI expansion from developing countries is stronger. Bilateral exchange rates promote China’s MPI to developed countries but are not significant in developing countries.

#### 4.5.2. Stage Heterogeneity

Table 10 compares estimates before and after 2013. In the post-2013 period, economic distance and value-chain distance become strongly negative and statistically significant, and network distance becomes more strongly negative as well. This suggests that, as global trade uncertainty rises and supply chains reorganize, structural proximity and network positioning increasingly condition China’s role as a destination market. Comparative advantage remains positive and significant in both periods. The estimated effect of FTAs is positive in the earlier period but weakens later, consistent with diminishing marginal trade-creation gains after initial liberalization effects are absorbed.

#### 4.5.3. Product Heterogeneity

Table 11 reports product-level heterogeneity. Several consistent patterns emerge. Comparative advantage is positive across all product groups, indicating a broad-based role of competitiveness in shaping China’s import-side market provision. Economic distance is more strongly negative for intermediate and consumer goods, suggesting that similarity in income and demand structure is particularly important for these categories. Value-chain distance is most salient for primary goods, where production structure and upstream positioning may play a larger role. Network distance is negative for most product groups, implying that greater destination concentration is associated with lower dependence on China’s market in primary, intermediate, and consumer goods. FTAs are positively associated with MPI across product categories and are especially strong for intermediate goods, consistent with trade agreements facilitating deeper integration in production-related imports.

## 5. Discussion

This study quantifies China’s import-side market provision and decomposes it into category (EM), quantity (QM), and price (PM) margins, then links their evolution to partner heterogeneity and gravity-type determinants. This section interprets what the margin patterns imply for how a large destination market expands and how exporter characteristics shape exposure to that market.

### 5.1. Interpretation of Margin Dynamics

Two features stand out. First, EM remains persistently high (above 0.9) and contributes little to MPI growth, implying that China’s import demand already overlaps with most world product categories for most partners. Once category coverage is mature, further expansion in destination-market provision is less likely to come from adding new categories and more likely to arise from deepening within existing categories [7]. Second, growth is primarily intensive: QM accounts for most of the long-run increase in MPI. In the most recent period, PM contributes a much larger share of MPI growth, suggesting a shift toward a quantity-price co-movement regime. Because unit values embed quality composition, contract terms, and short-run price shocks, the rising role of PM should be interpreted as stronger price-related dynamics rather than as unambiguous quality upgrading. Distinguishing these mechanisms will require more granular product attributes or firm-level import data.

### 5.2. Distribution Dynamics and Heterogeneity

Kernel-density evidence shows a rightward shift and a thicker right tail for MPI and QM, indicating widening dispersion in partner reliance on China’s market. A subset of exporters becomes substantially more China-oriented, while others remain less exposed. This pattern is consistent with network perspectives: Network distance is negative, indicating that exporters with more concentrated destination portfolios tend to rely less on China. Heterogeneity analyzes further suggest that China’s market provision is stronger for primary and intermediate goods and for developing exporters, largely through quantity deepening, while price-related dynamics are more pronounced in intermediate and consumer goods and among developed exporters. The gravity estimates are consistent with demand similarity and value-chain proximity mechanisms: larger economic distance and greater value-chain-position gaps are associated with weaker market provision [31,32]. Comparative advantage remains a robust positive correlate [54]. FTAs are associated with higher MPI; comparisons across the margin equations suggest that this association is mainly reflected in quantity deepening [23,44].

### 5.3. Limitations and Future Research

Several limitations qualify the interpretation. First, PM is based on unit values and cannot cleanly separate quality upgrading from price shocks, changes in product mix, or market power. Future work could combine product characteristics, quality-estimation methods, or firm-level customs data to decompose price dynamics more precisely. Second, the empirical design is reduced-form. Structural approaches could map changes in import-side market provision to welfare, resilience, and diversification outcomes for exporters, especially under global shocks and policy uncertainty. Third, annual data may mask short-run adjustments in prices and quantities. Higher-frequency data, where available, would help quantify the responsiveness of different margins to trade-policy changes, geopolitical events, and demand disruptions. Extending the same framework to other large destination markets (e.g., the United States or the European Union) would also help assess the generalizability of the “external market provider” concept. Fourth, our baseline and robustness specifications rely on log-linear OLS and Tobit estimation. While the Tobit model accounts for the bounded nature of MPI, alternative estimators such as Poisson Pseudo-Maximum Likelihood (PPML) or fractional response models could provide additional robustness for the MPI equation specifically [58]. However, because the decomposed margins QM and PM are unbounded by construction (they represent relative quantity and price ratios that can exceed 1), these bounded-outcome estimators cannot be uniformly applied across our four-equation system.

Future work could explore a hybrid estimation strategy that employs fractional response models for MPI and EM alongside OLS for QM and PM. Also the future research should extend the analytical framework to the post-2022 period in order to capture recent global developments, including supply-chain restructuring, RCEP implementation, and continued geopolitical trade tensions. Comparative application to other major importing economies such as the United States and the European Union would further enhance the generalizability of the proposed measurement framework, interpreted through an information-theoretic lens.

## 6. Conclusions

This paper analyzes China’s role as a global “external market provider” by measuring its external market share (MPI) and decomposing it into category, quantity, and price margins using the Hummels–Klenow tripartite framework. Using panel data covering 168 source countries over the period 2001–2022, the study documents distribution dynamics through kernel density estimation and identifies the determinants and heterogeneity of China’s import-side market provision using an extended gravity model. First, China’s external market share increased markedly over the sample period, while the category margin remained persistently high, indicating broad and stable product coverage of China’s import demand. However, the contribution of the category margin to market share growth is limited and becomes negative in recent years, suggesting that further expansion increasingly occurs through deepening within existing product categories. Second, the marginal decomposition reveals a structural transition from a quantity-dominated expansion model toward a volume–price co-movement model, with the price margin accounting for a growing share of market expansion in the post-2018 period. Third, China’s external market provision exhibits substantial heterogeneity across products, regions, and partner-country development levels, with stronger market provision for primary and intermediate goods and increasing dispersion in partner dependence over time.

The gravity analysis shows that China’s external market provision is shaped by demand similarity, value-chain proximity, comparative advantage, trade-network positioning, and trade agreements, with effects that are generally stronger for developing countries and for primary and intermediate goods. These findings remain robust across multiple specifications and robustness checks. Overall, by providing a margin-based and distribution-sensitive measurement of China’s external market provision, this paper contributes empirically to the literature on trade margins and bilateral trade structure and offers a demand-side perspective on how large destination markets influence the evolution of global trade patterns under conditions of rising uncertainty. Several avenues for future research merit attention. First, the measurement framework presented here can be readily applied to other large destination markets, such as the United States, the European Union, and India, to enable systematic cross-country comparisons of import-side market provision. Second, extending the temporal coverage to the 2023–2026 period would be particularly valuable, given the continued evolution of US–China trade tensions, the implementation effects of RCEP, ongoing supply-chain restructuring (e.g., “friendshoring” and “nearshoring” strategies), and the aftermath of the COVID-19 pandemic. These developments have likely induced further shifts in destination-portfolio entropy across source countries, which the HHI-based diversification framework, with its entropy interpretation, is well suited to capture. Third, future work could deepen the theoretical foundations by developing a structural model that endogenizes destination entropy and derives testable predictions for how entropy-linked diversification interacts with trade margins under different market structures.

## Figures and Tables

**Table 1:** Variance Inflation Factor (VIF) Diagnostics.

Variable	Description	VIF	Tolerance (1/VIF)
ECO	Economic proximity	2.14	0.467
GVC	GVC similarity	2.48	0.403
NET	Network diversification/entropy distance	2.76	0.362
TC	Trade costs	1.98	0.505
INS	Institutional factors	2.31	0.433
FTA	Free trade agreement	1.67	0.599
EXE	Export capability	2.45	0.408
GDP	Economic size control	2.12	0.472
Distance	Geographic distance	1.89	0.529
Mean VIF		2.20	

**Table 2:** Product Classification Matching Based on BEC Rev.4 and HS1996.

Type	BEC Code	Main Products and Quantities
Primary product	111, 21, 31	Primary food, industrial supplies, fuel, lubricants, etc., 4
Intermediate goods	121, 22, 322, 42, 53	Processing food and beverage, industrial supplies, capital goods and spare parts for transport equipment, etc., 284
Consumer goods	112, 122, 522, 61, 62, 63	Household consumption items, non-industrial transport equipment, (non-/semi-) durable goods, 1238 items
Capital goods	41, 521	Capital goods, industrial transport equipment, etc., 674

Note: This study excludes Type 7: Unclassified Special Transactions and Commodities from the BEC classification.

**Table 3:** China’s Share and Marginal Contribution Rate to the Global Market.

Metrics/Phase			Full Sample	2001–2012	2013–2018	2019–2022
Annual Average	MPI	(%)	10.52	8.16	12.48	14.64
	EM		0.92	0.91	0.92	0.92
	QM		0.10	0.08	0.12	0.13
	PM		1.13	1.10	1.14	1.21
Growth Rate (%)	MPI		5.98	9.58	2.39	1.50
	EM		0.10	0.22	0.16	−0.33
	QM		5.38	9.06	1.71	0.77
	PM		0.50	0.29	0.52	1.06
Contribution Rate (%)	MPI		100	100	100	100
	EM		1.70	2.34	6.78	−21.84
	QM		89.93	94.65	71.45	51.11
	PM		8.37	3.01	21.78	70.73

**Table 4:** China’s Share and Marginal Contribution Rate: Product Heterogeneity.

Product/Indicator			Primary	Intermediate	Consumer	Capital
Annual Average	MPI	(%)	16.57	11.42	4.28	8.88
	EM		0.93	0.93	0.92	0.94
	QM		0.17	0.10	0.04	0.08
	PM		1.03	1.22	1.24	1.16
Growth Rate (%)	MPI		6.40	4.53	8.95	4.09
	EM		0.35	0.11	0.44	0.00
	QM		6.56	3.26	6.97	3.52
	PM		−0.50	1.16	1.55	0.57
Contribution Rate (%)	MPI		100	100	100	100
	EM		5.42	2.34	4.88	0.05
	QM		102.46	72.02	77.85	85.99
	PM		−7.88	25.65	17.27	13.96

**Table 5:** China’s Share and Marginal Contribution Rate: Continental Heterogeneity.

Metrics			Asia	Europe	Africa	N. America	S. America	Oceania
Annual Avg.	MPI	(%)	14.89	4.05	17.12	5.73	15.33	22.97
	EM		0.95	0.89	0.78	0.95	0.78	0.92
	QM		0.14	0.03	0.22	0.05	0.20	0.25
	PM		1.13	1.27	0.98	1.17	0.96	0.97
Growth (%)	MPI		4.38	6.93	8.29	5.04	9.13	7.57
	EM		0.04	0.31	0.81	0.08	1.16	0.34
	QM		3.40	6.04	7.44	4.75	7.50	7.12
	PM		0.95	0.57	0.04	0.21	0.47	0.11
Contrib. (%)	MPI		100	100	100	100	100	100
	EM		0.85	4.54	9.75	1.66	12.74	4.45
	QM		77.54	87.22	89.80	94.24	82.15	94.10
	PM		21.60	8.24	0.45	4.10	5.11	1.45

**Table 6:** China Share and Marginal Contribution Rate: Country Heterogeneity.

Country	Annual Average				Growth Rate (%)				Contrib (%)		
	MPI	EM	QM	PM	MPI	EM	QM	PM	EM	QM	PM
Developed	9.56	0.95	0.08	1.18	4.89	0.00	4.14	0.75	−0.06	84.80	15.26
Japan	16.51	0.99	0.16	1.05	4.28	−0.09	3.87	0.50	−2.16	90.47	11.70
Korea	21.76	0.98	0.18	1.20	2.81	0.05	1.63	1.13	1.93	57.99	40.08
USA	6.72	0.98	0.06	1.20	5.08	0.02	4.22	0.85	0.43	82.94	16.63
Germany	5.21	0.97	0.04	1.30	6.14	0.03	5.39	0.72	0.53	87.78	11.69
Australia	24.16	0.93	0.26	0.97	7.37	0.36	6.99	0.01	4.89	94.94	0.18
Developing	12.14	0.85	0.13	1.07	6.87	0.65	5.92	0.30	9.51	86.19	4.30
Russia	8.85	0.85	0.10	1.06	6.07	0.79	6.59	−1.31	13.01	108.61	−21.63
Brazil	15.96	0.84	0.19	0.99	9.97	0.83	8.77	0.36	8.35	88.00	3.65
Indonesia	10.81	0.93	0.12	0.99	7.71	0.35	7.82	−0.46	4.49	101.43	−5.92
Saudi Arabia	10.97	0.93	0.12	0.97	8.76	0.00	9.73	−0.97	0.01	111.06	−11.07
Vietnam	10.53	0.94	0.09	1.25	3.12	0.49	−2.05	4.68	15.85	−65.84	149.98

Note: Only the top five import sources from developed and developing countries in 2022 are reported.

**Table 7:** Benchmark Regression Results.

Variable	MPI	EM	QM	PM
${ECO}_{ijt}$	−0.017 * (0.009)	−0.027 (0.025)	−0.022 *** (0.002)	−0.019 (0.033)
${GVC}_{ijt}$	−0.085 ** (0.039)	0.024 (0.083)	−0.268 *** (0.088)	0.183 (0.143)
${TC}_{ijt}$	0.059 *** (0.010)	0.161 *** (0.020)	0.168 *** (0.019)	0.187 *** (0.051)
${NET}_{ijt}$	−0.035 *** (0.012)	−0.094 *** (0.026)	−0.027 ** (0.011)	0.090 ** (0.041)
${INS}_{ijt}$	0.00400 (0.004)	0.022 ** (0.009)	0.005 (0.009)	−0.023 (0.021)
${EXE}_{ijt}$	−0.001 (0.003)	−0.016 (0.015)	−0.011 (0.009)	−0.018 * (0.009)
${FTA}_{ijt}$	0.027 *** (0.007)	0.011 (0.017)	0.024 ** (0.009)	−0.012 (0.022)
Constant	0.131 *** (0.046)	0.588 *** (0.127)	0.502 *** (0.113)	0.859 *** (0.204)
N	3026	3026	3026	3026
$R^{2}$	0.902	0.875	0.729	0.702

Note: Controls for country and year fixed effects. Clustered robust standard errors in parentheses. *, **, *** denote significance at 10%, 5%, and 1% levels.

**Table 8:** Endogeneity Treatment and Robustness Tests.

Variable	Model 1	Model 2	Model 3	Model 4	Model 5
${ECO}_{ijt}$	−0.017 * (0.010)	−0.006 (0.008)	−0.003 (0.011)	−0.003 * (0.002)	−0.018 ** (0.009)
${GVC}_{ijt}$	−0.093 ** (0.046)	−0.102 *** (0.031)	−0.035 (0.023)	−0.041 ** (0.021)	−0.088 ** (0.039)
${TC}_{ijt}$	0.042 *** (0.008)	0.057 *** (0.008)	0.067 *** (0.009)	0.048 *** (0.009)	0.059 *** (0.009)
${NET}_{ijt}$	−0.021 * (0.011)	−0.036 *** (0.012)	−0.017 (0.013)	−0.016 ** (0.006)	−0.034 *** (0.012)
${INS}_{ijt}$	0.004 (0.004)	0.004 (0.004)	−0.001 (0.003)	−0.002 (0.002)	0.004 (0.004)
${EXE}_{ijt}$	−0.002 (0.004)	−0.000 (0.002)	−0.004 (0.003)	0.001 (0.001)	−0.001 (0.003)
${FTA}_{ijt}$	0.028 *** (0.008)	0.027 *** (0.007)	−0.001 (0.005)	0.019 ** (0.009)	0.027 *** (0.007)
N	2895	2777	3026	3027	3026
$R^{2}$	0.870	0.921	-	-	0.903

Note: In this table, the asterisks (*, **, ***) denote the statistical significance levels of the coefficient estimates, based on *p*-values from hypothesis tests: * Significant at the 10% level (*p* < 0.10); ** Significant at the 5% level (*p* < 0.05); *** Significant at the 1% level (*p* < 0.01). The numbers in parentheses below each coefficient represent the robust standard errors, which measure the uncertainty or variability of the estimated coefficients. A smaller standard error relative to the coefficient value indicates a more precise estimate.

**Table 9:** Correlates: Country-Specific Heterogeneity (MPI).

Variable	Developed	Developing
${ECO}_{ijt}$	−0.010 (0.006)	−0.018 ** (0.007)
${GVC}_{ijt}$	−0.047 (0.029)	−0.084 * (0.045)
${TC}_{ijt}$	0.006 (0.005)	0.064 *** (0.010)
${NET}_{ijt}$	0.012 (0.010)	−0.032 ** (0.013)
${INS}_{ijt}$	−0.001 (0.002)	−0.001 (0.006)
${EXE}_{ijt}$	0.002 ** (0.001)	0.023 (0.017)
${FTA}_{ijt}$	0.008 * (0.004)	0.027 *** (0.009)
N	792	2234
$R^{2}$	0.984	0.898

Note: The symbols *, **, and *** denote the statistical significance levels of the estimated coefficients, based on *p*-values from hypothesis testing: * Significant at the 10% level (*p* < 0.10); ** Significant at the 5% level (*p* < 0.05); *** Significant at the 1% level (*p* < 0.01). Numbers in parentheses below each coefficient represent robust standard errors, measuring the precision of coefficient estimates (smaller values indicate higher precision).

**Table 10:** Correlates: Stage Heterogeneity (MPI).

Variable	2001–2013	2013–2022
${ECO}_{ijt}$	−0.319 (0.257)	−1.246 *** (0.383)
${GVC}_{ijt}$	−0.053 (0.042)	−0.114 *** (0.042)
${TC}_{ijt}$	0.279 *** (0.045)	0.244 *** (0.041)
${NET}_{ijt}$	−0.019 (0.032)	−0.105 *** (0.039)
${INS}_{ijt}$	−0.008 (0.012)	−0.008 (0.008)
${EXE}_{ijt}$	−0.064 (0.102)	−0.338 (0.425)
${FTA}_{ijt}$	0.013 ** (0.006)	0.009 (0.011)
N	1463	1246
$R^{2}$	0.965	0.973

Note: The symbols **, and *** denote the statistical significance levels of the estimated coefficients, based on *p*-values from hypothesis testing: ** Significant at the 5% level (*p* < 0.05); *** Significant at the 1% level (*p* < 0.01). Numbers in parentheses below each coefficient represent robust standard errors, measuring the precision of coefficient estimates (smaller values indicate higher precision).

**Table 11:** Correlates: Product Heterogeneity (MPI).

Variable	Primary	Intermediate	Consumer	Capital
${ECO}_{ijt}$	−0.018 (0.013)	−0.012 ** (0.005)	−0.015 ** (0.007)	−0.008 (0.007)
${GVC}_{ijt}$	−0.146 *** (0.046)	−0.043 (0.032)	−0.033 (0.025)	0.013 (0.024)
${TC}_{ijt}$	0.010 * (0.006)	0.064 *** (0.008)	0.096 *** (0.013)	0.070 *** (0.010)
${NET}_{ijt}$	−0.070 *** (0.012)	−0.021 ** (0.010)	−0.018 ** (0.009)	0.001 (0.003)
${INS}_{ijt}$	0.004 (0.005)	−0.001 (0.003)	0.003 (0.003)	−0.001 (0.002)
${EXE}_{ijt}$	−0.004 (0.006)	−0.012 (0.009)	−0.009 (0.007)	0.006 ** (0.002)
${FTA}_{ijt}$	0.020 * (0.011)	0.072 *** (0.007)	0.014 * (0.008)	0.021 *** (0.007)
N	2943	3003	2965	2856
$R^{2}$	0.906	0.832	0.774	0.704

Note: The symbols *, **, and *** denote the statistical significance levels of the estimated coefficients, based on *p*-values from hypothesis testing: * Significant at the 10% level (*p* < 0.10); ** Significant at the 5% level (*p* < 0.05); *** Significant at the 1% level (*p* < 0.01). Numbers in parentheses below each coefficient represent robust standard errors, measuring the precision of coefficient estimates (smaller values indicate higher precision).

## Data Availability

The raw trade data used in this study are from the China Free Trade Zone Service Network (see Section 3.4). The replication dataset (constructed indicators and the processed country–year panel used to generate the tables and figures) is publicly available on Zenodo: https://doi.org/10.5281/zenodo.18477144 (access on 1 April 2026).
